# Stakeholder Experiences in Participatory Mental Health Model Development: A Focus Group Study

**DOI:** 10.1111/hex.70810

**Published:** 2026-08-13

**Authors:** Maartje Vriens, Hendrika J. Valkenburg, Judith E. Bosmans, Silke Gabbert, Dwayne Meijnckens, Talitha L. Feenstra, G. Ardine de Wit, EVAneMENT study team

**Affiliations:** ^1^ Centre for Public Health, Healthcare & Society National Institute for Public Health and the Environment Bilthoven the Netherlands; ^2^ Department of Health Sciences, Faculty of Science, Vrije Universiteit Amsterdam Amsterdam Public Health research institute Amsterdam the Netherlands; ^3^ Department of Statistics, Data Science and Modelling National Institute for Public Health and the Environment Bilthoven the Netherlands; ^4^ Centre for Economic Evaluations & Machine Learning, Trimbos Institute Netherlands Institute of Mental Health and Addiction Utrecht the Netherlands; ^5^ Centre for Safety of Substances and Products National Institute for Public Health and the Environment Bilthoven the Netherlands; ^6^ Department of Knowledge Innovation & Research, MIND Amersfoort the Netherlands; ^7^ Groningen Research Institute of Pharmacy, Faculty of Science and Engineering University of Groningen Groningen the Netherlands

**Keywords:** computational model development, evaluation, focus groups, mental health, participatory modelling, stakeholder involvement

## Abstract

**Background:**

The involvement of stakeholders in computational model development is an emerging and promising practice that can enhance the understanding of the model context and improve its relevance for supporting decision‐making processes. However, little is known about stakeholders’ experiences in these engagement processes. This study explored the experiences of stakeholders on their involvement in the participatory development of a youth mental health model.

**Methods:**

Focus groups were conducted with four stakeholder groups: (1) experts by experience who have experienced mental health problems, (2) young adults aged 16–25 interested in mental health, (3) professionals working in (preventive) mental health services for youth and (4) youth mental health researchers. Fourteen focus groups and one individual interview were conducted across three distinct rounds, in which a total of 43 stakeholders participated. Additionally, questionnaires were administered to evaluate the focus group sessions. The data were analysed using thematic content analysis.

**Results:**

During the focus groups, participants valued comparing ideas across stakeholder groups and incorporating lived experiences into model development, but expressed doubts about the validity of their contributions and noted insufficient transparency in the modelling process. Participants also encountered complexities related to specific requirements of modelling, including the need to simplify the concept of mental health and to address interactions among mental health factors.

**Conclusion:**

Tailored information provision per stakeholder group and transparency of the modelling process can help address challenges in participatory modelling. This study offers practical guidance to improve stakeholder contributions in computational model development, ultimately improving model quality and supporting policy‐making in complex domains such as mental health.

**Patient or Public Contribution:**

A participatory research approach was used to develop the computational model, thereby ensuring that it incorporated lived experiences and priorities of the stakeholders. Additionally, the involvement of two researchers with lived experience and expertise in patient participation in mental health research, together with an advisory board of advocates with lived experience, facilitated the continuous integration of these stakeholder group perspectives throughout all phases of the research process.

## 1 Introduction

The rising global prevalence of mental health problems and the substantial contribution of mental ill‐health to the global burden of disease have made the improvement of population mental health an important global public health priority [1, 2, 3]. They frequently emerge in childhood and young adulthood, with severe conditions such as depression and anxiety affecting approximately one in four individuals before the age of 25 [4]. Preventive interventions have shown to be cost‐effective to improve health outcomes [5, 6, 7], yet selecting the most impactful option while remaining within budget is challenging for decision‐makers due to the wide variety of available options [7, 8]. Computational models, computer‐based frameworks designed to simulate real‐world situations, can be used to estimate the potential costs and benefits of policy options prior to their implementation, supporting informed decision‐making on the optimal resource allocation to achieve the greatest impact on outcomes [9, 10, 11, 12]. Developing such a model begins with a conceptual model that outlines the key factors and interactions within the system [13].

To enhance system understanding, various stakeholders can be engaged in the model development process through an approach known as participatory modelling [14, 15, 16, 17]. This approach is rooted in the broader concept of public and patient involvement (PPI), which emphasises the importance of including patients and members of the public in research to ensure that real‐world perspectives are considered [18, 19, 20]. While PPI focuses primarily on patients and the public, participatory modelling is a practical application of PPI that builds on this concept by also involving additional stakeholders, such as healthcare professionals, who have a vested interest in the model. In participatory computational modelling, these stakeholders actively contribute to different stages of computational model development, including system mapping, conceptual model development, intervention identification and prioritisation, and model validation [21]. The inclusion of stakeholder perspectives from the early stages of model development remains relatively uncommon, as input is typically solicited only after computational models have been developed [22, 23]. However, incorporating stakeholders’ knowledge and contextual insights from the onset of model development is expected to increase the model's acceptability among decision‐makers and other end‐users, and to result in models that are more credible and valid than those developed solely by a modelling team [14, 15, 16, 17, 21, 22, 23].

Despite these advantages, participatory computational modelling presents challenges for both the modelling team and the stakeholders, as bringing together individuals with diverse backgrounds and levels of technical expertise introduces unique ethical challenges, particularly given the technical complexities of computational model development [21, 24, 25, 26]. One major challenge is effectively navigating group power dynamics by ensuring that all stakeholders can participate equitably and respectfully, regardless of their familiarity with computational modelling and their level of technical knowledge. Other challenges include enabling less technically experienced stakeholders to understand and engage with the modelling process, and deciding which activities stakeholders can contribute to [21, 24, 25, 26, 27, 28].

While some guidelines exist to address the challenges associated with participatory computational modelling [29, 30, 31, 32], they are often focused on the modelling teams’ perspectives and lack detailed consideration of how stakeholders experience the participatory computational modelling process. Insights into these experiences are crucial for refining and optimising stakeholder engagement methods, as they can identify potential barriers and highlight opportunities for achieving more inclusive and effective collaborations. Although several participatory modelling studies have been conducted, they often conclude with the development of a qualitative conceptual model (e.g. 33, 34, 35, 36, 37), leaving unaddressed the unique complexities involved in developing a functional computational model. Only one previous study, conducted by Freebairn et al. [38], explored stakeholder experiences within participatory computational modelling processes.

Therefore, the aim of this study is to explore stakeholders’ experiences of their involvement in a participatory computational modelling study focused on youth mental health.

## 2 Methods

In this participatory modelling study, a conceptual model was developed with stakeholders that will serve as the basis for a computational model designed to estimate the long‐term health effects of preventive policies on youth mental health in the Netherlands (ages 12 – 25) [13, 39]. Focus groups were conducted to facilitate interaction among participants during the sessions [40]. The focus groups aimed to identify the key factors and relationships influencing the development of mental health problems in later life that should be incorporated into a computational model for mental health. Simultaneously, during the focus groups, we also examined the experiences of participants involved in the participatory modelling process. A more comprehensive description of how stakeholders were involved in the modelling process, along with detailed information on the model development and the integration of stakeholder input into the model, is provided elsewhere [39]. To conduct this research, a study team was assembled consisting of eleven researchers with diverse areas of expertise, including qualitative research methodologies, health economics, modelling, and policy evaluation, as well as two researchers with both lived experience of mental health problems and expertise in patient participation in mental health research. To ensure thorough documentation of the reporting for this study, we adhered to the Consolidated Criteria for Reporting Qualitative Studies (COREQ), with the corresponding checklist provided in Appendix A [41].

### 2.1 Participants

A stakeholder mapping exercise was conducted in collaboration with members from the study team to identify potentially relevant stakeholder groups [42]. The following four stakeholder groups were included: (1) experts by experience who have experienced mental health problems during their youth and can reflect on these problems, (2) young adults aged 16 – 25 interested in mental health, (3) professionals working in (preventive) mental health services for youth, and (4) researchers in the field of youth mental health. We initially also aimed to include policymakers involved in national and local mental health policy development, yet when we began inviting them, several indicated that the objectives of the sessions did not align with their roles and that they lacked sufficient expertise in mental health factors to contribute meaningfully. Therefore, we conducted a separate study with both local and national policymakers (*n* = 28) involved in youth mental health policy development [43]. Participants from the four stakeholder groups were recruited through purposive sampling, utilising the networks of the study team members. Additionally, we employed the social media platform of MIND, a Dutch organisation that advocates for individuals with (emerging) mental health problems, to recruit experts by experience and young adults interested in mental health [44]. All participants were approached through email.

### 2.2 Procedure

Focus groups, each lasting 1.5 h, were conducted across three distinct rounds. All participants initially invited to the first round were subsequently invited to participate in the following rounds. Multiple focus group sessions were conducted per round to foster a safe environment and minimise the potential effect of power imbalances, as participants from similar stakeholder groups could be included within the same session. Additionally, organising multiple sessions facilitated the comparison of results across groups. Furthermore, conducting multiple rounds enabled participants to reflect on the evolving models, and to refine and expand their own contributions over time. During the first round, focus groups were composed of participants from the same stakeholder group. In the subsequent two rounds, focus groups either included a mix of researchers and professionals, or a mix of experts by experience and young adults. Additionally, in the second round, an individual interview was conducted with a researcher who was unable to attend the scheduled focus group sessions. The individual interview adhered to the same design and structure employed in the focus group sessions. Focus groups with researchers and professionals were conducted online using Microsoft Teams [45]. In‐person sessions with experts by experience and young adults were held, offering an online participation option for participants unable to attend physically. Each focus group was facilitated by two early‐career researchers (MV, HV), with a third study team member present: either a senior researcher (AW, TF, BW) or a lived‐experience researcher (DM, PU) to provide additional information on the study. To address potential power dynamics between academic and non‐academic stakeholders, senior researchers participated minimally in focus groups with researchers and professionals, contributing only when clarification on the modelling study was needed. Lived‐experience researchers attended sessions with experts by experience and young adults to promote a safe and supportive environment. Prior to the focus groups, participants were provided with preparatory materials, including a summary of the anticipated modelling study and additional information regarding the focus group content (Appendix B). All focus group materials were piloted with fellow researchers, with a specific emphasis on avoiding complex language. Additionally, the materials were reviewed by an advisory board consisting of advocates for individuals with lived experience of mental health problems, and a scientific advisory board composed of researchers that have experience in mental health modelling.

Each session of each round started with participants introducing themselves to ensure familiarity with each other's background, followed by a presentation on the background of the study. Afterwards, we discussed topics related to key mental health factors and their relationships, as well as topics addressing participants’ reflections and experiences on their involvement in the participatory modelling process. An overview of all topics discussed and activities conducted during and after the focus groups is provided in Appendix C. At the start of the in‐person focus group sessions, experts by experience and young adults provided written information on their motivations for participating in this study. Additionally, upon the conclusion of those sessions, experts by experience and young adults were reimbursed for their travel costs and received a gift voucher as compensation for their participation. Upon the completion of the third‐round focus group sessions, participants were invited to provide written feedback via an online questionnaire, addressing both the final design of the model and the participatory modelling process. Additionally, in a separate questionnaire, an evaluation form was distributed in which participants were asked to rate their level of agreement with various statements regarding the structure and atmosphere of the focus group sessions, as well as their personal role and contributions during the discussions. Participants were also invited to provide detailed elaborations on these topics and to offer suggestions for enhancing future focus group sessions.

### 2.3 Data Analysis

All focus groups and the individual interview were audio recorded and transcribed verbatim. The transcripts were subsequently anonymised to ensure participant confidentiality. Each transcript was independently coded by two researchers (MV, HV), using ATLAS.ti Web software [46]. Any discrepancies in coding were resolved through discussion between the coders. The data analysis adhered to the principles of thematic content analysis as outlined by Braun and Clarke [47]. An iterative approach of inductive coding was employed to identify themes from the focus group data. A similar methodological approach was used to code and analyse the information obtained from the questionnaires, which was conducted independently from the focus group data. The coding framework used for the analysis of the focus group data is presented in Appendix D, and for the analysis of the questionnaire data in Appendix E.

### 2.4 Ethical Considerations

All participants received both written and verbal information about the study prior to providing informed consent for the inclusion of their contributions in the research. Furthermore, participants were explicitly informed of their right to withdraw from the study at any time without the need to provide a justification. Before commencing the study, we obtained a statement from the Centre for Clinical Expertise of the Dutch National Institute for Public Health and the Environment, indicating that our study did not fall under the Dutch law for Medical Research Involving Human Subjects (WMO), and was therefore exempted from full ethical review.

## 3 Results

In total, fourteen focus groups and one individual interview were conducted between October 2023 and July 2024, involving 43 distinct stakeholders, 19 of whom participated in two or more sessions. An overview of the number and types of stakeholders participating in each focus group round is presented in Table 1.

**Table 1:** Overview of the number and types of stakeholders in each focus group round.

Focus group rounds	Number of stakeholders				Total
	Professionals	Researchers	Experts by experience	Young adults	
Round 1	12	10	8	7	37
Round 2	6	6	6	3	21
Round 3	4	1	3	4	12

This section outlines four themes and fifteen subthemes that emerged from the focus group data (Table 2), which are illustrated using verbatim quotes.

**Table 2:** Overview of main themes and subthemes that emerged from the focus group data.

Experienced strengths related to the process of participatory modelling	Experienced challenges related to the process of participatory modelling
Compare ideas across stakeholder groups	Unclarities related to the study
Sense of ownership over the model	Doubts about validity of own and other participants’ contributions
Incorporate lived experiences	Insufficient transparency in modelling process
Enjoyment in focus group participation	

Encountered complexities related to computational modelling	Encountered complexities related to modelling mental health
Need for simplification	Interactions between mental health factors
Making future projections	Influence of context and culture
Initial framework for ongoing model development	Variability within and between individuals
	Measurement of mental health

### 3.1 Theme 1: Experienced Strengths Related to the Process of Participatory Modelling

Subtheme 1 Compare ideas across stakeholder groups

This theme was more often expressed by experts by experience and young adults, though the other groups also reported that a key strength of the participatory modelling process was its ability to facilitate the comparison of ideas both within and across stakeholder groups, ultimately enhancing the quality of the model's content.

Expert by experience, R1: *‘We are just a small subgroup of the group after all. I am curious to see what other people you will encounter in your research, and what insights that will yield.’*

Subtheme 2 Sense of ownership over the model

Participation in the focus groups gave predominantly experts by experience and young adults a sense of ownership over the final conceptual model. By engaging in multiple focus group rounds, participants indicated feeling genuinely involved in the modelling process, were able to observe the model's development, and could recognise their contributions from previous rounds, which in turn strengthened their sense of ownership.

Expert by experience, R3: *‘If you have a predefined framework already, where all that is left is to add the stamp: ‘Oh, an experience expert should have looked at that, well, you can observe for an hour’ and then the least irritating points are included in the study. But here, we have really built it together.’*

Subtheme 3 Incorporate lived experiences

The possibility to incorporate lived experiences into model development was only identified by experts by experience as a strength of the participatory modelling process. They highlighted the added value of integrating practical examples and experiences into the modelling study concerning specific mental health topics, ultimately making the model more robust.

Expert by experience, R1: *‘For example, if I look at myself, I have experienced bullying in high school. And I just notice now, that I have been quite shaped by it, not necessarily in terms of memories, but in how I handle certain things, and I sometimes struggle with it.’*

Subtheme 4 Enjoyment in focus group participation

Predominantly experts by experience and young adults reported deriving enjoyment from their participation in the focus groups, which was attributed to their appreciation for the opportunity to observe the research process, reflect on mental health topics, and engage in discussions about these topics with other participants.

Young adult, R2: *‘I really enjoyed thinking along like this, and it was very interesting to see how your research process develops.’*

### 3.2 Theme 2: Experienced Challenges Related to the Process of Participatory Modelling

Subtheme 1 Unclarities related to the study

Participants described various unclarities concerning different aspects of the study. First, predominantly researchers and professionals expressed unclarities concerning the design of the study, including the study purpose, overall scope, the phase of their involvement in the modelling process, and the process for developing new versions of the conceptual model, specifically the sources and inputs used in its development. Second, primarily experts by experience and young adults expressed uncertainties regarding the activities expected of them during the focus groups. Finally, unclarities about the modelling details were mainly mentioned by researchers and professionals, who perceived computational modelling as a complex process. While some participants were uncertain about the potential of such models to enhance mental health, others did not understand how the model could project future mental health outcomes.

Professional, R1: *‘And this is where I get momentarily lost, and I wonder: how? It is not that I am saying the research is not worthwhile, but rather: how can you create a model that is predicting this [mental health]? That is really just due to my lack of scientifically informed knowledge.’*

Subtheme 2 Doubts about validity of own and other participants’ contributions

Some participants expressed doubts concerning the validity of their own contributions, questioning whether they could offer valuable insights on certain questions that were posed. Furthermore, predominantly researchers were sceptical about the extent to which focus group discussions could contribute to the modelling process. They highlighted that, while each viewpoint substantially influences the model development process, certain contributions may not represent the entire population.

Researcher, R2: *‘Have you been able to compare the focus group outcomes with what is described in the literature, is there any information about a possible hierarchy or effect size? These [mental health] factors are mentioned by Dutch experts, healthcare professionals, and the target group themselves, but is this also supported by what you found in the literature?’*

Subtheme 3 Insufficient transparency in modelling process

During the second and third rounds, researchers and professionals perceived insufficient transparency in the modelling process, particularly regarding the decisions made by the study team on the model structure discussed during the focus groups. They noted that insufficient background information provided regarding these decisions, along with limited transparency about how stakeholder input was incorporated in model development, restricted their participation in the sessions.

Researcher, R3: *‘On the one hand: this is a lot of information and it provides a clear overview of the model you have developed. But sometimes I miss a bit of the background, for example what aspects you got from the literature, that explain why you have made certain choices.’*

### 3.3 Theme 3: Encountered Complexities Related to Computational Modelling

Subtheme 1 Need for simplification

The need to simplify the concept of mental health was considered a complexity of computational modelling by all stakeholder groups, with researchers and professionals expressing this theme most frequently. They noted that defining a scope for the model, which involved making decisions concerning the specific age group to focus on, the mental health factors to be included, and the outcome measures to be modelled, required prioritising certain aspects of mental health, which came along with the exclusion of factors and contexts they considered important for a comprehensive understanding of mental health.

Researcher, R2: ‘*I also understand the difficulty, because it [mental health] is just very complex, and to unravel all that, that is quite a challenge. […] Does this really capture a big part of the picture?*’

Nonetheless, some participants acknowledged the necessity for simplification, suggesting that the computational model must be manageable to prevent it from becoming excessively confusing.

Young adult, R3: *‘It [model] has to remain manageable. Otherwise, it is actually not useful, because then it would just become a mess. As you have all these different aspects included, you might no longer know where to start.’*

Subtheme 2 Making future projections

A common theme among researchers and professionals was the complexity of having discussions that required making future projections regarding mental health factors. They reported difficulty in anticipating how these factors will evolve over time and how these would impact mental health.

Researcher, R1: *‘The question is, of course, what happens now at that age and how that carries over into later life. That is also quite difficult to know.’*

Subtheme 3 Initial framework for ongoing model development

All stakeholder groups emphasised the need for the computational model to function as an initial framework for ongoing model development and future research. They advocated for regular review and update of the model to assess whether additional factors should be incorporated in the future.

Professional, R3: *‘I think it [model] could be a very nice start. Yet, I think there are still some complications. I assume you will also continue to evaluate: what can be improved? I think it would be super valuable then.’*

### 3.4 Theme 4: Encountered Complexities Related to Modelling Mental Health

Subtheme 1 Interactions between mental health factors

Predominantly researchers and professionals described the large number of factors influencing mental health, along with the dynamic interactions among these factors, as a complexity of modelling mental health. They explained that all factors can influence each other, including potential indirect effects mediated through other factors, thereby forming a complex system of interactions that underlies the development of mental health problems.

Researcher, R2: *‘What is cause and what is effect? Again, I find it very difficult to distinguish between the two. […] Something can be a result of mental health problems, but it can also be a precursor to more severe mental problems.’*

Subtheme 2 Influence of context and culture

A common theme among all stakeholder groups was the important influence of the context in which mental health factors occur and the associated challenge of incorporating this influence into the development of a mental health model in the Dutch context. They reported that contexts can differently influence the development of mental health factors, thereby affecting the potential emergence of mental health problems. Additionally, participants noted that cultural differences among individuals can affect perceptions of mental health problems and approaches to address these within a cultural context.

Expert by experience, R2: *‘I think a bit of culture can also play a role here. I believe we are all raised differently, based on diverse cultural norms and values, which can definitely influence what your home environment looks like and what types of conflicts occur at home. And often, how much is mental health talked about, how much openness is there?’*

Subtheme 3 Variability within and between individuals

All stakeholder groups discussed the variability in how mental health factors are expressed both within and between individuals as a complexity of modelling mental health. Concerning the intra‐individual variability, they described that the influence of certain mental health factors can change over time and that the impact of these factors varies with age.

Professional, R1: *‘What constitutes a risk [for mental health problems] can also change over time, in my opinion. I think it is quite possible that something considered a risk at a young age is no longer a risk 5 years later.’*

Additionally, participants reported considerable inter‐individual variability, noting that individuals vary and cope differently with potential mental health problems.

Researcher, R1: *‘Losing a job or having to repeat a year at school can be stressful for one person, while for another it can be the beginning of a long‐awaited opportunity and a new chance.’*

Subtheme 4 Measurement of mental health

This final theme was primarily identified by researchers, although the other three groups also recognised the measurement of mental health as a complexity of developing a mental health model. They highlighted a lack of sufficient data on mental health and noted that certain factors are challenging to measure. Additionally, participants described the difficulty of defining mental health in a precise and comprehensive manner that fully captures the entire concept.

Researcher, R2: *‘Mental health is incredibly complex, especially since we still do not fully agree on exactly what mental health is.’*

### 3.5 Evaluation of Focus Groups

Next to the evaluations provided during the focus groups, 11 participants submitted written evaluations of the focus group sessions. This section summarises the themes uniquely identified in the evaluation data, with themes highlighted in italics. A more detailed overview of the evaluation results is available in Appendix F. Appendix G presents an overview of the additional questionnaire results, including information on participants’ motivations for engaging in this modelling study and their reflections on the participatory modelling process.

#### 3.5.1 Structure and Atmosphere

Participants identified several strengths of the focus groups, including the creation of a *comfortable and safe atmosphere*, *diverse group compositions throughout sessions*, and *the function of the focus groups as a platform for facilitating stakeholder interaction and collaboration*. The *clear structure of the sessions* was also noted as a strength, though some participants remarked *insufficient facilitator guidance*. Lastly, a few participants highlighted the *challenge of having meaningful discussions on mental health*, due to the inherent complexity arising from the broad nature of mental health.

#### 3.5.2 Personal Role and Contributions

Differing perspectives were expressed by participants regarding the possibility to provide their contributions during sessions. Although some noted *difficulties in providing input during discussions*, others felt that *all participants were given the opportunity to contribute*. Furthermore, some participants indicated that they *felt stakeholder input was used in model development*, while others expressed a *lack of clarity on how their input was used in model development*.

#### 3.5.3 Suggestions to Improve Focus Groups

Participants recommended *increasing the duration of focus group sessions*, *incorporating possibilities to provide written elaborations after the sessions*, and *adopting a less structured format* to allow for more in‐depth discussions on mental health. Additionally, participants suggested that the *evaluation of the process should have been conducted earlier*. Some participants reported difficulty recalling details of the focus groups due to the elapsed time, noting that 2 months had passed since the most recent session and that, for those who participated only in the first round, approximately 1 year had elapsed.

## 4 Discussion

Involving stakeholders in computational modelling is a promising but still underexplored approach. Developing models collaboratively can be challenging because of technical complexity involved and the varying levels of expertise among stakeholders. This study provides valuable insights into how stakeholders experienced taking part in a participatory mental health modelling study and offers practical guidance for future participatory modelling studies.

### 4.1 Main Insights From the Results

Stakeholders encountered various complexities throughout the modelling process, many of which arose from the inherent tension between the demand to adequately capture the multifaceted and highly complex nature of mental health, and the requirement for simplicity to ensure practical implementation of the model. The study team faced a similar dilemma in determining the type and amount of information to provide to stakeholders, aiming to ensure that stakeholders possess sufficient understanding to engage meaningfully in the process while minimising cognitive overload arising from excessive perceived complexity. However, several stakeholders frequently indicated during focus group sessions that they needed more information on several aspects of the study, which may have limited their ability to fully participate in the process.

Notable differences in experiences among stakeholder groups were observed, indicating that focus group experiences varied across groups despite the standardised session design. Researchers and professionals more frequently reported challenges and complexities related to both computational modelling and modelling mental health, whereas experts by experience and young adults more often emphasised the strengths of the participatory modelling process. These differences may reflect the specific roles for which stakeholders were invited to participate. For example, researchers were primarily included for their ability to provide a critical perspective based on scientific expertise, whereas experts by experience contributed valuable insights derived from their personal involvement with the topic.

### 4.2 Comparison With Literature

To date, limited studies have evaluated stakeholders’ experiences with their involvement in computational modelling processes [21]. Although there is one study available by Freebairn et al. [38] that explored stakeholder experiences across several participatory modelling studies [48, 49, 50, 51], their work focused exclusively on decision makers as the stakeholder group. Similar to our findings, Freebairn et al. [38] reported that stakeholders valued the co‐production inherent to participatory approaches. Notably, they identified a unique strength in that stakeholders placed significant value on the ability to observe how their input was integrated into the model, which improved understanding of the modelling process, enhanced transparency, and fostered trust in the model as a reliable decision‐support tool.

When designing a participatory research approach, using a framework to support stakeholder involvement throughout the research process can help ensure that stakeholders have meaningful opportunities to contribute [52]. Such frameworks may include those based on community‐based participatory research, which address considerations including the researcher‐participant relationships, and including stakeholders in the research design and dissemination of the research [53]. Other frameworks emphasise values such as respect, transparency, and inclusivity, which are essential for effective stakeholder engagement [52].

Stakeholder participation in research exists along a spectrum of engagement levels, with the International Association for Public Participation defining five levels, ordered from least to most engaged: informing, consulting, involving, collaborating, and empowering [54, 55]. In this study, some aspects of the research process took place at the collaborate level, as two researchers with lived experience participated as equal partners within the study team throughout all phases of the research process. Yet, stakeholder engagement during the focus groups primarily occurred at the involve level, wherein stakeholders collaborated directly with the study team to provide their inputs into the modelling process, although final decision‐making authority remained with the study team. We acknowledge the potential benefits of more actively engaging stakeholders and recognise that engaging all stakeholders at the collaborate level could have strengthened the participatory nature of our research. Such a higher level of participation might have led to different experiences of stakeholders regarding their engagement. Yet, engaging stakeholders without research experience requires adjustments to researchers’ responsibilities and shifts in power dynamics between researchers and stakeholders. These must be carefully managed to prevent imbalances not only between researchers and stakeholders but also within stakeholder groups, as stakeholders may vary widely in their research experience and persuasive power [28, 56, 57, 58, 59]. Our findings revealed that stakeholders experienced uncertainties about the study and sometimes perceived themselves unable to contribute to the research process meaningfully, which is a common theme in patient and public involvement in research in general, where insufficient preparation or an overload of tasks among stakeholders can lead to challenges in participation [18, 19]. Furthermore, our findings highlighted instances of conflicting perspectives among stakeholders and concerns regarding the validity of contributions from other stakeholders, with some emphasising the need to validate focus group results against existing scientific literature. This underscores both the importance and challenge of ensuring the legitimacy of stakeholder involvement in participatory research, where it is essential to recognise that stakeholders’ experiential knowledge is relevant, valuable, and essential for informing and enriching the research context [60]. Transitioning to higher levels of stakeholder participation, such as collaborating or empowering, requires researchers to acknowledge stakeholders’ legitimacy and commit to decentralising decision‐making power, while stakeholders have to commit to active participation and remain open to differing perspectives.

### 4.3 Strengths and Limitations

A notable strength of our study was the inclusion of experts by experience as a key stakeholder group, who provided valuable insights into their experiences with participatory modelling, a perspective often underrepresented in modelling initiatives due to barriers such as extensive time commitments and concerns about stigmatisation [61]. Additionally, to facilitate a safe and supportive environment and mitigate power differences during the focus groups with experts by experience and young adults, stakeholder groups that often have less experience in research, lived‐experience researchers attended the sessions instead of senior researchers. Another strength is that the study benefited from the participation of 43 individuals from different stakeholder groups in interactive sessions designed to facilitate meaningful interactions between diverse stakeholders. Lastly, we ensured the continuous integration of stakeholder group perspectives throughout both the design and interpretation of the research by involving two researchers with lived experience and expertise in patient participation in mental health research, in addition to consulting an advisory board of advocates with lived experience.

Several limitations should be acknowledged as well. First, although we involved a broad range of stakeholders, the inclusion of national and local policymakers could have provided further insights into how they inform and experience participatory modelling. Although initially invited, policymakers declined to participate and were therefore involved in a separate study [43]. A second limitation is the potential for self‐selection bias, as participants may have been highly motivated and particularly interested in mental health modelling, potentially resulting in more positive experiences. Yet, our sample also included critical stakeholders, as most findings highlight the challenges and complexities encountered. Third, our study had a relatively high drop‐out rate, which may be attributed to fixed scheduling of focus group sessions, necessary given the large number of stakeholders and the logistics of organising multiple rounds. Although we did not systematically collect reasons for non‐participation, time constraints was most frequently cited. The inclusion of experts by experience and young adults may have further contributed to drop‐out, as these groups can be more challenging to retain and occasionally cancelled at short notice. A fourth limitation is the absence of a theoretical framework to guide the exploration of participant experiences. Applying frameworks such as Stakeholder Theory [62] or the Social Ecological Model [63] could have provided additional structure for interpreting stakeholder views and understanding the influences of individual, social, and organisational factors. Fifth, a substantial time gap between the final focus group sessions and the moment at which the written evaluation was conducted may have contributed to fewer stakeholders completing the evaluation form. In retrospect, distributing the evaluation form immediately after each session would have likely enhanced stakeholders’ recall of the sessions and increased engagement with the evaluation process. Lastly, the transferability of the study findings may be somewhat limited, as the study specifically focused on model development in mental health in a Dutch setting, which may differ from other contexts with unique complexities. Nonetheless, many aspects of stakeholder involvement identified in this study, including the experienced strengths and challenges, are likely comparable and relevant to other participatory modelling contexts as well, especially when the final aim is the development of a computational model.

### 4.4 Lessons Learned and Recommendations

As outlined in the introduction, some guidelines are available to assist researchers in the implementation of participatory modelling process [24, 28, 29, 30, 31, 32]. In addition, reflection on our own participatory computational modelling activities has yielded further lessons and recommendations that can offer practical guidance for future modelling studies seeking to involve stakeholders.

Variations in experiences and preferences across different stakeholder groups underscore the importance of tailoring focus group materials to each group, especially regarding the level of detail. To accommodate this, comprehensive preparatory materials should be provided to all stakeholders prior to the sessions, while essential information should also be briefly summarised during the sessions. These materials may be tailored through the involvement of stakeholder group representatives during the research design phase, as they possess insights into stakeholder needs while also maintaining an understanding of the project objectives to be obtained. Additionally, offering an optional pre‐session conversation, allowing stakeholders to access further information and pose questions about the process, may enhance their understanding and engagement.

Synthesising inputs from multiple sessions into a single contribution for the subsequent iteration introduced challenges. Some stakeholders had difficulty recognising their specific contributions and perceived a lack of transparency in the modelling process. While organising multiple sessions is considered valuable for fostering a supportive environment for all types of stakeholders, including those facing greater challenges in contributing, greater emphasis should be directed toward enhancing clarity and ensuring stakeholders understand how their inputs are integrated into the model. This transparency could be achieved by providing a comprehensive report after each focus group round, detailing the specific inputs received and describing how the study team has incorporated these contributions into further development of the model.

Another insight derived from the focus groups was that some stakeholders perceived a lack of flexibility during the sessions. They emphasised that mental health is a complex topic, requiring adequate time and flexibility for in‐depth discussions. Stakeholders suggested that extending the duration of focus group sessions and adopting a less structured format could provide greater opportunities for meaningful engagement and comprehensive discussions.

Lastly, stakeholders highlighted several strengths of their involvement in the modelling process, also noting that the focus groups provided value beyond model development by acting as a platform for collaboration and interaction among diverse stakeholders and organisations. This underscores the broader impact of stakeholder involvement in research, demonstrating its dual role in contributing to the research process and facilitating meaningful collaboration and knowledge exchange among stakeholders.

## 5 Conclusion

Engaging stakeholders into computational modelling processes is a developing practice that is underexplored. This study provides valuable insights into stakeholder experiences in a participatory modelling study focused on mental health and offers practical recommendations for future participatory modelling studies. Tailored information provision and continued transparency on the modelling process, including decisions made by the study team based on stakeholder input, are essential for effective stakeholder participation. Given the inherent complexity of modelling mental health, integrating diverse stakeholder perspectives can substantially enhance the understanding of the context for the study team and improve its relevance for supporting decision‐making processes. By applying the insights gained to improve participatory modelling processes, stakeholder contributions can be more effectively used to improve the quality and relevance of computational models, ultimately supporting better‐informed policy‐making in complex domains such as mental health.

## Author Contributions

**Maartje Vriens:** conceptualisation, data curation, formal analysis, investigation, methodology, resources, software, visualisation, writing – original draft, writing – review and editing. **Hendrika J. Valkenburg:** conceptualisation, data curation, formal analysis, investigation, methodology, resources, software, validation, writing – review and editing. **Judith E. Bosmans:** conceptualisation, methodology, supervision, writing – review and editing. **Silke Gabbert:** conceptualisation, methodology, supervision, writing – review and editing. **Dwayne Meijnckens:** conceptualisation, investigation, methodology, resources, validation, writing – review and editing. **Talitha L. Feenstra:** conceptualisation, funding acquisition, investigation, methodology, project administration, supervision, writing – review and editing. **G. Ardine Wit:** conceptualisation, funding acquisition, investigation, methodology, project administration, supervision, writing – review and editing.

## Funding

This research was funded by the Strategic Program of the Netherlands National Institute for Public Health and the Environment, grant number S/133032/01. The funder did not influence the design and execution of this research.

## Conflicts of Interest

The authors declare no conflicts of interest.

## Supporting information


Supporting File

## Data Availability

Data available on request due to privacy/ethical restrictions.
