# Supplementary material for: Stakeholder Experiences in Participatory Mental Health Model Development: A Focus Group Study
Source: Health Expect. 2026 Aug 13;29(4):e70810. doi: 10.1111/hex.70810 (PMC13470604; doi:10.1111/hex.70810)
Supplement: Supplementary file 1 [file HEX-29-e70810-s001.docx]

**Supporting information – overview**

[Appendix A: Consolidated Criteria for Reporting Qualitative Research	2](#_Toc225939082)

[Appendix B: Overview of preparatory materials for the focus groups	4](#_Toc225939083)

[Appendix C: Comprehensive overview of focus group activities	7](#_Toc225939084)

[Appendix D: Coding framework used for data analysis of focus groups and questionnaires	10](#_Toc225939085)

[Appendix E: Detailed overview of focus group evaluations	12](#_Toc225939086)

[Appendix F: Results additional questionnaires	15](#_Toc225939087)

[References used in appendices	16](#_Toc225939088)

# **Appendix A: Consolidated Criteria for Reporting Qualitative Research (1)**

| **Domain, Items and Guide Questions/Descriptions** | **Reported on Page No.** |
| --- | --- |
| **Domain 1: Research team and reflexivity** | |
| ***Personal characteristics*** | |
| **Interviewer/facilitator** – Which author/s conducted the interview or focus group? | Page 6 |
| **Credentials** – What were the researcher’s credentials? *e.g. PhD, MD* | Page 6 |
| **Occupation** – What was their occupation at the time of the study? | Page 6 |
| **Gender** – Was the researcher male or female? | N/A |
| **Experience and training** – What experience or training did the researcher have? | Page 6 |
| ***Relationships with participants*** | |
| **Relationship established** – Was a relationship established prior to study commencement? | Page 5 |
| **Participant knowledge of the interviewer** – What did the participants know about the researcher? *e.g. personal goals, reasons for doing the research* | Page 5 |
| **Interviewer characteristics** – What characteristics were reported about the interviewer/facilitator? *e.g. bias, assumptions, reasons and interests in the research topic* | Page 5 |
| **Domain 2: Study design** | |
| ***Theoretical framework*** | |
| Methodological orientation and Theory – What methodological orientation was stated to underpin the study? *e.g. grounded theory, discourse analysis, ethnography, phenomenology, content analysis* | Page 7 |
| ***Participant selection*** | |
| **Sampling** – How were participants selected? *e.g. purposive, convenience,*  *consecutive, snowball* | Page 5 |
| **Method of approach** – How were participants approached? *e.g. face-to-face, telephone, mail, email* | Page 5 |
| **Sample size** – How many participants were in the study? | Page 7 |
| **Non-participation** – How many people refused to participate or dropped out? Reasons? | Supporting information page 7 |
| ***Setting*** | |
| Setting of data collection – Where was the data collected? *e.g. home, clinic, workplace* | Page 6 |
| Presence of non-participants – Was anyone else present besides the participants and researchers? | Page 6 |
| Description of sample – What are the important characteristics of the sample? *e.g. demographic data, date* | Page 7 |
| ***Data collection*** | |
| Interview guide – Were questions, prompts, guides provided by the authors? Was it pilot tested? | Pages 5 – 6 |
| Repeat interviews – Were repeat interviews carried out? If yes, how many? | Page 7 |
| Audio/visual recording – Did the research use audio or visual recording to collect the data? | Page 7 |
| Field notes – Were field notes made during and/or after the interview or focus group? | Pages 5 – 6 |
| Duration – What was the duration of the interviews or focus group? | Page 5 |
| Data saturation – Was data saturation discussed? | N/A |
| Transcripts returned – Were transcripts returned to participants for comment and/or correction? | N/A |
| **Domain 3: Analysis and findings** | |
| ***Data analysis*** | |
| Number of data coders – How many data coders coded the data? | Page 7 |
| Description of the coding tree – Did authors provide a description of the coding tree? | Page 6 & supporting information pages 10 – 11 |
| Derivation of themes – Were themes identified in advance or derived from the data? | Page 7 |
| Software – What software, if applicable, was used to manage the data? | Page 7 |
| Participant checking – Did participants provide feedback on the findings? | Supporting information pages 7 – 9 |
| ***Reporting*** | |
| Quotations presented – Were participant quotations presented to illustrate the themes/findings? Was each quotation identified? *e.g. participant number* | Pages 8 – 14 |
| Data and findings consistent – Was there consistency between the data presented and the findings? | Pages 8 – 14 |
| Clarity of major themes – Were major themes clearly presented in the findings? | Pages 8 – 14 |
| Clarity of minor themes – Is there a description of diverse cases or discussion of minor themes? | Pages 8 – 14 |

Information from: Tong A, Sainsbury P, Craig J. Consolidated criteria for reporting qualitative research (COREQ): a 32-item checklist for interviews and focus groups. International Journal for Quality in Health Care. 2007;19(6):349-57.

# **Appendix B: Overview of preparatory materials for the focus groups**

Participants were provided with preparatory materials prior to the focus groups, which included 1) a summary of the modelling study and 2) information about the focus group content. The complete set of materials is presented below.

**1. Summary of modelling study: Computational model for mental health in youth**

This research project aims to develop a computational model that will allow us to project the mental health of youth in the Netherlands over a 30-year time horizon. Using this model, we will be able to estimate the potential long-term effects of policies by comparing scenarios with and without those policies.

Background and objective of the research

At present, an increasing number of youth in the Netherlands are experiencing mental health problems. A range of new preventive policies is being introduced to improve the mental health of youth and to prevent potential mental disorders later in life. However, we currently lack the ability to accurately estimate the long-term impacts of different policy options on overall mental health outcomes. Yet, this information is essential for making well-informed decisions.

The objective of this study is, therefore, to develop a model capable of projecting the long-term effects of policy interventions on mental health outcomes.

Research design

This focus group focuses on identifying the most relevant factors of mental health among youth aged 12 to 25 years, which may contribute to the development of mental disorders later in life (18-35 years). Additionally, we aim to identify the relationships between these factors. Six to eight participants will take part in this interview.

**2. Information on focus group content: initial list with mental health factors and their definitions (2)**

| **Factor** | **Definition** |
| --- | --- |
| **Health & lifestyle** | |
| Alcohol use | Consumption of alcohol, including frequency, quantity, and/or problematic use. |
| Drug use | Consumption of drugs, including frequency, quantity, and/or problematic use. |
| Perceived health | A health measure covering the individual’s perception of all relevant health aspects. |
| Physical activity | All bodily movements and sports during leisure time, as well as during work, study, commuting, and household activities. |
| Sleep | Sleep behaviour, including aspects such as quantity and quality of sleep. |
| Smoking | Tobacco use by an individual, including frequency, quantity, and/or problematic use. |
| **Social** | |
| Education level | The highest education a person has completed and the opportunities available to achieve it. |
| Employment status | An individual's position in the labour market (e.g., employed, unemployed, looking for work, or not in the labour force). |
| School or study demands | (Perceived) demands of school or study on a student, such as academic pressure, long school days, homework control, curriculum variety, and unclear instructions or assignments. |
| Social environment at school/studies | (Perceived) quality of social connections and relationships at school, including school atmosphere, support from teachers or mentors, bullying, conflicts, or inappropriate behaviour. |
| Social environment at work | (Perceived) quality of social connections and relationships at work, such as workplace atmosphere, support from colleagues or supervisors, conflicts, or inappropriate behaviour. |
| Social living environment | (Perceived) quality of social connections and relationships in an individual's immediate living environment, including having a social network, family and friend support, and cyberbullying. Note: Social safety of the living environment is excluded here and categorized under "Safe living environment." |
| Work (including side jobs) demands | (Perceived) demands of work on the employee, such as work pressure, long hours, task control, job variety, and role ambiguity. |
| **Family & yourself** | |
| Body image | The extent to which a person is satisfied with their physical appearance and how they generally perceive their looks. |
| Home environment | Domestic or private circumstances, such as household composition, physical and mental health of family members, poverty, conflicts, housing quality, and household safety. |
| Parental upbringing | Factors determining a child’s upbringing within the household, such as parenting style, attachment, and the quality of the parent-child relationship. |
| Physical living environment | Physical aspects and amenities in an individual's living area, such as environment and traffic, presence of nature or water, urbanisation, built living environment, and leisure opportunities. |
| Safe living environment | Perceived or actual (un)safety in the immediate living environment, such as neighbourhood crime and violence, and perceptions thereof, including social safety in the area. |
| Social media use | Use of online platforms for social interaction, including frequency, quantity, and/or problematic use. |
| Traumatic experiences | Unresolved past events such as accidents, the death of an important person, sexual abuse, or violence. |
| **Life in general** | |
| Livelihood security | The degree to which a person feels assured of having resources to meet basic life needs, such as financial, housing, and income security, and the social security system. |
| Participation in society | Involvement in social activities such as school and work, volunteering, relationships, and politics. |
| Quality of life | A person’s perception of their life position within the context of the culture and value system in which they live, as well as their goals, expectations, standards, and interests. |
| Societal norms and values | (Perceived) quality of interaction standards and principles in the Dutch society, including discrimination, (perceived) stigmatisation, or performance pressure. |

# **Appendix C: Comprehensive overview of focus group activities**

This appendix provides a detailed overview of 1) the number of sessions conducted in each focus group round, 2) the period over which the sessions occurred, 3) the number of stakeholders who were involved, 4) the objectives that were addressed, and 5) the activities performed during the three rounds of focus groups. Furthermore, it includes a comprehensive overview of the activities conducted after the focus group sessions had concluded.

**Focus group round 1**

**Number of sessions**: 8, with 2 sessions for each stakeholder group.

**Period**: October and November 2023

**Number of stakeholders**: 37* (professionals: 12, researchers: 10, experts by experience: 8, young adults: 7)

* During one of the sessions in the first round, a participant expressed strong criticism of the research and refused to continue participation during the session. Following a mutual agreement between the research team and the participant, it was concluded that further involvement in subsequent rounds would be inappropriate, and the participant was therefore not invited to participate in subsequent rounds.

**Objective**: Identify key mental health factors in youth (12-25 years) that most influence the development of mental disorders at a later age (until 40 years), and explore potential relationships between these factors.

**Activities**:

1) Introductions and study overview: Participants introduced themselves, followed by a presentation providing background information on the study.

2) Rating mental health factors: Participants individually rated the degree of influence (on a scale of 1 to 5) of 24 mental health factors (that were included in an initial overview) in youth (12-25 years) on the development of mental disorders later in life (until 40 years). They were also invited to suggest any important mental health factors missing from the initial overview.

3) Selection of influential factors: The medians of the ratings for each mental health factor were calculated. Factors with a median score higher than 4 were selected for further discussion in the next activity.

4) Identifying relationships between factors: Participants worked as a group to identify relationships between the most influential mental health factors, also in relation to the development of mental disorders (such as depression or anxiety disorders). Participants could add any missing factors to the discussion.

5) Feedback on participatory modelling process and reflection: Participants were provided with an overview of the steps taken to develop the new version of the conceptual model, and key points discussed during the session were summarised. All participants were invited to reflect on these steps, to reflect on the activities conducted, and to share their experiences with these activities. Additionally, participants were asked whether they felt their input from the previous round had been incorporated into the design of the conceptual model. The next steps in the study were also outlined.

6) Motivation for participation (for experts by experience and young adults only): Participants were asked to individually write down their motivation for participating in this study.

**Focus group round 2**

**Number of sessions**: 5, with 3 sessions for professionals and researchers combined and 2 sessions for experts by experience and young adults combined. Furthermore, the sessions consisted of 4 focus groups and 1 individual interview.

**Period**: February and March 2024

**Number of stakeholders**: 21 (professionals: 6, researchers: 6, experts by experience: 6, young adults: 3)

**Objective**: Explore how the selected mental health factors can be made more operational and concrete for inclusion in a computational model, and address any follow-up questions regarding unclear aspects from the first focus group round.

**Activities**: 1) Introductions and study overview: Participants introduced themselves, followed by a presentation providing background information on the study.

2) Overview of results from round 1: Participants were presented with a summary of the results from all sessions conducted during focus group round 1. They were invited to reflect on these results.

3) Splitting up factors: The study team created an initial categorisation of key mental health factors identified in focus group round 1 to make them more operational and concrete. Participated were asked to discuss whether any underlying factors were missing or if they agreed with the initial categorisation.

4) Follow-up questions: Participants were asked follow-up questions to clarify any unclear aspects from focus group round 1. Additionally, they were invited to provide their perspectives on additional factors that were identified during focus group round 1, which participants felt were missing from the initial overview.

5) Feedback on participatory modelling process and reflection: Participants were provided with an overview of the steps taken to develop the new version of the conceptual model, and key points discussed during the session were summarised. All participants were invited to reflect on these steps, to reflect on the activities conducted, and to share their experiences with these activities. Additionally, participants were asked whether they felt their input from the previous round had been incorporated into the design of the conceptual model. The next steps in the study were also outlined.

**Focus group round 3**

**Number of sessions**: 2, with 1 session for professionals and researchers combined and 1 session for experts by experience and young adults combined.

**Period**: July 2024

**Number of stakeholders**: 12 (professionals: 4, researchers: 1, experts by experience: 3, young adults: 4)

**Objective**: Present and discuss the penultimate version of the conceptual model and gather feedback on participants’ perspectives concerning the participatory development process.

**Activities**: 1) Introductions and study overview: Participants introduced themselves, followed by a presentation providing background information on the study.

2) Overview of results from rounds 1 and 2: Participants were presented with a summary of the results from all sessions conducted during focus group rounds 1 and 2. They were invited to reflect on those results.

3) Feedback on participatory modelling process: Participants were provided with an overview of the steps taken to develop the penultimate version of the conceptual model and were invited to reflect on these steps. They were also asked whether they felt their input from previous rounds had been incorporated into the penultimate version of the model.

4) Feedback on design model: Participants were asked to provide feedback on the penultimate version of the conceptual model, as well as on its overall usability.

5) Summary and reflection: The key points discussed during the session were summarised, and all participants were asked to reflect on the activities conducted and their experiences with these activities. The next steps in the study were also outlined.

**After focus group sessions: written feedback**

**Period**: September and October 2024

**Number of stakeholders**: 7 (professionals = 4, researchers = 2, experts by experience = 1,

young adults = 0)

**Objective**: Enable participants to offer detailed feedback on the final version of the conceptual model and share their insights on the participatory modelling process.

**Activities**: 1) Overview presentation: Participants received a comprehensive written overview of all steps involved in developing the final version of the conceptual model.

2) Feedback on design model and participatory modelling process: Participants were invited to provide written feedback on the final design of the conceptual model, offer feedback on the participatory modelling process, and indicate whether they felt they could contribute to the development of the model.

**After focus groups sessions: participant evaluation**

**Period**: September and October 2024

**Number of stakeholders**: 11 (professionals = 3, researchers = 3, experts by experience = 5,

young adults = 0)

**Objective**: Enable participants to evaluate their experiences with the process of participating in the modelling study after the conclusion of the focus groups.

**Activities**: 1) Evaluation of focus group experiences on structure and atmosphere: Participants were asked to assess their level of agreement with statements related to structure and atmosphere of the focus group sessions. They were also prompted to explain their responses and identify the strengths and challenges they experienced during the sessions.

2) Evaluation of focus group experiences on personal role and contributions: Participants were asked to asses their level of agreement with statements related to their personal role and contributions during the focus group sessions. They were encouraged to explain their responses and suggest circumstances that would improve their ability to provide input during future sessions.

# **Appendix D: Coding framework used for data analysis of focus groups and questionnaires**

This appendix provides an overview of the included themes and subthemes in the data analysis, with subthemes underlined and, where necessary, accompanied by explanatory information.

**Table 1: Coding framework used for data analysis of focus groups**

| **Themes** | **Subthemes** |
| --- | --- |
| Experienced strengths related to the process of participatory modelling | Compare ideas across stakeholder groups both within and across stakeholder groups |
|  | Sense of ownership over the model |
|  | Incorporate lived experiences of the participants into model development |
|  | Enjoyment in focus group participation |
| Experienced challenges related to the process of participatory modelling | Unclarities related to the study about study design, study activities that had to be conducted during focus groups and modelling details on how computational modelling works |
|  | Doubts about validity of own and other participants’ contributions |
|  | Insufficient transparency in modelling process |
| Encountered complexities related to computational modelling | Need for simplification to simplify concepts included in the model |
|  | Making future projections about factors when developing a computational model |
|  | Initial framework for ongoing model development to have the possibility to include additional factors in the model in the future |
| Encountered complexities related to modelling mental health | Interactions between mental health factors |
|  | Influence of context and culture on the development of mental health factors |
|  | Variability within and between individuals can influence the development of mental health factors |
|  | Measurement of mental health |

The second coding framework presented outlines the data analysis of the questionnaires.

Themes newly identified in the questionnaire data are highlighted in blue, whereas themes previously identified in the focus group data are presented in black.

**Table 2: Coding framework used for data analysis of questionnaires**

| **Themes** | **Subthemes** |
| --- | --- |
| **Stakeholder motivations for participation in the modelling study** | |
| Motivations for participation in process | Contribute to improvement of mental health |
|  | Share personal experiences |
|  | Gain more insights into topic of mental health |
|  | Address societal stigma around mental health |
| **Stakeholder reflections on the participatory modelling process** | |
| Reflections on the final design of the conceptual model | Model incompleteness |
|  | Model completeness |
|  | Need for simplification |
|  | Interactions between mental health factors |
|  | Measurement of mental health |
|  | Influence of context and culture |
|  | Initial framework for ongoing model development |
|  | Appreciation for substantial efforts in developing model |
| Reflections on the process of participatory modelling | Doubts about validity of other participants’ contributions |
|  | Lack of transparency in modelling process |
|  | Unclarity about study design |
|  | Sense of involvement in the modelling process |
|  | Enjoyment in focus group participation |
| **Stakeholder evaluations of the focus group sessions** | |
| Evaluations of structure and atmosphere of the focus groups | Comfortable and safe atmosphere |
|  | Diverse group compositions throughout sessions |
|  | Function of focus groups as platform for interaction and collaboration |
|  | Clear structure of focus groups |
|  | Insufficient facilitator guidance |
|  | Insufficient background information on the study |
|  | Challenging to have meaningful discussions on mental health |
| Evaluations of personal role and contributions | Possibility for all participants to contribute |
|  | Feeling that stakeholder input was used in model development |
|  | Difficulty to provide input during discussions |
|  | Lack of clarity on how participant input was used in model development |
|  | Doubts about validity of other participants’ contributions |
| Suggestions to improve the focus groups | Increase duration of focus group sessions |
|  | Possibility to provide written elaborations after focus group sessions |
|  | Adopt less structured format |
|  | Conduct evaluation earlier in process |

# **Appendix E: Detailed overview of focus group evaluations**

Eleven participants provided written evaluations of the focus group sessions. The information provided by the participants has been synthesised, with the identified themes presented in italics below.

*Evaluations of structure and atmosphere of the focus groups*

Overall, participants perceived the focus groups as both useful and enjoyable, with a clear structure, as reflected by the majority of responses indicating “strongly agree” or “somewhat agree”. However, responses were more diverse regarding the extent to which participants felt they were able to participate and whether their contributions were taken seriously, with some participants selecting the response of “somewhat disagree”. The detailed responses on the statements are presented in Figure 1.

Figure 1: Overview of participant responses from the evaluation form on statements concerning structure and atmosphere

Several strengths and challenges were identified regarding the structure and atmosphere of the focus groups. One of the key strengths reported by participants was the creation of a *comfortable and safe atmosphere*, which was fostered by small group sizes that facilitated the opportunity for all participants to engage in the discussions. Other strengths that were highlighted included the *diverse group compositions throughout multiple sessions*, which encouraged engaging discussions and broadening stakeholder perspectives, as well as the *function of the focus groups as a platform for stakeholder interaction and collaboration*. Participants also reported the *clear structure of the focus groups* as a strength, with each session initiating with a comprehensive explanation of the discussion topics and being led by facilitators who were well-organised. On the other hand, some participants remarked that the focus groups occasionally had *insufficient facilitator guidance*, resulting in chaotic sessions characterised by reduced focus. Another challenge that was commonly mentioned was the *insufficient provision of background information on the study*, with notable lacking information concerning the study’s objectives, processes, criteria for expert selection, and integration with existing literature. Lastly, some participants highlighted the *challenge of having meaningful discussions on mental health*, due to the inherent complexity arising from the broad nature of mental health.

*Evaluations of personal role and contributions*

In general, participants indicated that there was sufficient space to present their input and that they considered themselves successful in providing input during the sessions, with responses ranging from “strongly agree” to “somewhat agree”. However, slightly lower levels of agreement were reported concerning the extent to which their contributions were taken seriously and adequately reflected in the final result of the group discussions; the conceptual model. The specific responses on the statements are illustrated in Figure 2.

Figure 2: Overview of participant responses from the evaluation form on statements concerning their personal role and contributions during the focus groups

Participants provided detailed reflections concerning their personal role and contributions within the focus groups. A reflection noted by some participants was the *difficulty to provide input during discussions*, where the combination of the occasional intensity of the discussions and the expectation for contributions to be primarily oral posed difficulties for less outspoken individuals. Conversely, other participants reported feeling that *all participants were given the opportunity to contribute*. Another reflection of some participants concerned the *lack of clarity about how participant input has been used* *in model development*, although others indicated that they *felt* *stakeholder input was used in the model development*. Lastly, some participants expressed *doubts about the validity of other participants’ contributions*, with concerns that not all participants were sufficiently capable of providing valid contributions.

*Suggestions to improve the focus groups*

Participants were invited to provide suggestions for improving the focus groups. One commonly cited recommendation was to *increase the duration of focus group sessions*, which would allow for more extensive discussions on mental health topics and enable participants to introduce themselves more comprehensively. Additionally, some participants expressed a preference for the *possibility to provide* *written elaborations* *following the focus group sessions* to further articulate their viewpoints. Another suggestion included *adopting a less structured format* to create additional space for mental health discussions. Lastly, participants suggested that the *evaluation of the process should have been conducted earlier*. Some participants reported difficulty recalling details of the focus groups due to the elapsed time, noting that two months had passed since the most recent session and that, for those who participated only in the first round, approximately one year had elapsed. Some participants suggested that the evaluation could be conducted immediately after each focus group round or concurrently during the session itself.

# **Appendix F: Results additional questionnaires**

Fifteen participants provided information concerning their motivations for participating in this modelling study and seven participants offered reflections on the participatory modelling process. The information provided by the participants has been synthesised, with the identified themes presented in italics below.

**Stakeholder motivations for participation in the modelling study**

Experts by experience and young adults have provided information concerning their motivations to participate in the modelling study. The primary motivations cited were 1) the aspiration to *contribute to the improvement of mental health* and 2) the possibility to *share their personal experiences* to support others. Further motivations for participation included 3) an interest in *gaining deeper insights into mental health* by examining factors contributing to the development of mental health problems and 4) an effort to *address the societal stigma associated with mental health problems* to promote open dialogue.

**Stakeholder reflections on the participatory modelling process**

*Reflections on the final design of the conceptual model*

Participants frequently highlighted the perceived *incompleteness of the model* due to the absence of important factors and contexts related to mental health, although a smaller group considered the *model complete* (8 vs. 3 times described respectively). Another frequently reported theme on the final design was the *need for simplification* in model development. Additional reflections were related to the complexity of representing mental health in the model, attributed to *interactions between mental health factors*, the challenges associated with the *measurement of mental health*, and the critical *influence of the context and culture* on the development of mental health factors. Participants also noted the importance for the model to serve as an *initial framework for ongoing model development* that allows for the inclusion of additional factors in the future. Lastly, participants expressed *appreciation for the substantial efforts* invested in developing the final design of the conceptual model.

*Reflections on the process of participatory modelling*

Themes that were primarily reported in the reflections of participants were the *doubts about the validity of other participants’ contributions* and the *lack of transparency in the modelling process*. Furthermore, participants noted some *unclarities* related to the *study design*. Lastly, participants expressed a *sense of involvement in the modelling process* and indicated their *enjoyment* in participating.

# **References used in appendices**

1. Tong A, Sainsbury P, Craig J. Consolidated criteria for reporting qualitative research (COREQ): a 32-item checklist for interviews and focus groups. International Journal for Quality in Health Care. 2007;19(6):349–57.

2. Valkenburg HJ, Vriens M, Meijnckens D, Rodenburg J, Wiering DS, Wijnen BFM, de Wit GA, Feenstra TL. Conceptualization of a decision-analytic model in youth mental health: an application of stakeholder engagement in model development in the Netherlands. Expert Review of Pharmacoeconomics & Outcomes Research. 2026;26(2):221–30.
